# Association of health behaviors with function and health-related quality of life among patients with Parkinson’s disease

**DOI:** 10.1186/s13584-023-00588-3

**Published:** 2024-01-03

**Authors:** Michal Duvdevani, Galit Yogev-Seligmann, Ilana Schlesinger, Maria Nassar, Ilana Erich, Rafi Hadad, Michal Kafri

**Affiliations:** 1 Department of Occupational Therapy, Faculty of Social Welfare & Health Sciences, University of Haifa, Mount Carmel, POB 3338, 3103301 Haifa, Israel; 2 Movement Disorders Institute, Department of Neurology, Rambam Health Care Campus, Haifa, Israel; 3 Technion Faculty of Medicine, Haifa, Israel; 4 Department of Physical Therapy, Faculty of Social Welfare & Health Sciences, University of Haifa, Haifa, Israel

**Keywords:** Health behaviors, Parkinson’s disease, Patient activation, Structural equation modeling, Health related quality of life

## Abstract

**Background:**

Current evidence on chronic conditions favors promotion of health behaviors as a mean to positively impact health outcomes. In Parkinson’s disease, performing health behaviors is indicated as a means to fight the long-lasting burden of the disease. Understanding actual engagement in health behaviors and patient activation and their association to function and health-related quality of life is therefore important. Our objectives were, among people with Parkinson’s disease: (1) to characterize health behaviors including utilization of rehabilitative treatments, physical activity, and patient activation levels, and (2) to test the associations between these health behaviors and health outcomes.

**Methods:**

A cross-sectional study of 88 people with Parkinson’s disease (age 66.84 ± 8.8) was conducted. Participants answered questionnaires measuring health behaviors including utilization of health professions treatments, physical activity, patient activation, and health outcomes consisting of function and health-related quality of life. Linear regression models were conducted to test associations between measured health behaviors, function and health-related quality of life.

**Results:**

Participants rarely engage in rehabilitative treatments, but showed high levels of patient activation. Controlled by demographics and disease severity, physical activity and patient activation were associated with function (b = 0.41, *p* < .001; b = 0.2, *p* = .02, respectively) and physical activity but not patient activation, which was associated with health-related quality of life (b = 0.19, *p* = .03). There was also interaction effects of physical activity and non-motor symptoms, and physical activity and motor symptoms on health-related quality of life (b = 0.19, *p* = .02 and b = − 0.22, *p* = .01, respectively).

**Conclusions:**

In respect to their potential health-related benefits for people with Parkinson’s disease, health professionals’ treatments are underutilized. Findings supported the importance of health behaviors for maintaining function and health-related quality of life among people with Parkinson’s disease. They also show a differential contribution of motor and non-motor symptoms to the association between physical activity and quality of life. It is suggested that policy makers encourage opportunities for physical activity tailored for people with Parkinson’s disease and adopt a proactive stance towards enhancing awareness and use of rehabilitation services.

*Trial registration* NCT05211700, ClinicalTrials.gov ID: NCT05211700 first release 12/30/2021, https://classic.clinicaltrials.gov/ct2/show/NCT05211700

**Supplementary Information:**

The online version contains supplementary material available at 10.1186/s13584-023-00588-3.

## Introduction

Parkinson’s disease (PD) is an incurable, chronic, progressive neurodegenerative disorder that affects motor, cognitive, and autonomic functions [1, 2]. The number of patients living with PD (PwP) is poised for growth due to aging of the world’s population [3]. Because the life expectancy of PwP is only slightly shorter than that of the general population [4, 5], PwP foresees managing symptoms for an extended duration, including severe disability and its associated medical complexities [6–8]. Given the increasing impact of PD on the economy, healthcare systems, and patients themselves, there’s a pressing need to facilitate strategies that can be widely used in diverse PwP populations. The current evidence on chronic conditions favors promotion of health behaviors [9–11] as a means of positively impacting health outcomes.

At the individual level, health behaviors refer to any activity the patient performs to get well (e.g., receiving treatment from medical providers, lifestyle adjustments) and to the individual’s beliefs and perceptions related to health maintenance, restoration, and improvement [12]. In PD, non-pharmacological treatment approaches focus on everyday life functioning (e.g., gait, falls, activities of daily living, speech) and their underlying impaired body functions (e.g., balance, dexterity). Therefore, utilization of multidisciplinary rehabilitative treatments, which should include physical therapy, occupational therapy, speech and language pathology and psychology, is recommended [13, 14]. Several systematic reviews and meta-analyses published in recent years concluded that rehabilitative treatments such as physical therapy have significant effects on motor symptoms and function, including balance, gait, risk of falls, and freezing of gait [15–17]. Reports of physical therapy insurance claims, however, range from 14 to 60% in different countries [18].

In addition, physical activity plays a major role in the non-pharmacological treatment of PwP, and its effects are widely discussed in the context of brain plasticity, cognitive function, motor symptoms and physical capacity [19–22]. Long-term engagement in physical activity, is therfore an important lifestyle-related health behavior.

The concept of patient activation captures the mental aspects of health behaviors: people’s knowledge, skills, and confidence in managing their health. Higher patient-activation levels indicate higher readiness to adopt behaviors that maintain or improve health. Compared with people with low levels of activation, people with high activation levels are more likely to adopt health behaviors such as maintaining physical activity [23]; Thus, they are more likely to experience better health outcomes [24, 25].

Previous studies reported the effect of structured health profession interventions or physical activity on different health domains in PwP, and showed the positive role these activities play in maintaining health outcomes [26–30]. These studies do not, however, represent actual health behaviors performed by PwP in real life (i.e., without structured intervention). Our goal in the current study was to measure health behaviors related to PD effectively performed by PwP in real life, and to test the relationship between these behaviors and health outcomes. More specifically, our objectives were, among PwP living in the community: (1) to characterize self-reported utilization of rehabilitative treatments, self-reported engagement in physical activity, and patient activation levels; (2) to test the possible associations between these aspects of health behaviors and health outcomes, including function and health-related quality of life (HRQoL).

We hypothesized that greater utilization of rehabilitative treatment, greater engagement in physical activity and higher patient activation will be associated with better function and HRQoL.

## Methods

Study design: a cross-sectional study with a convenience sampling.

### Study sample

A total of 88 PwP were recruited from the Movement Disorders Institute, Department of Neurology, Rambam Health Care Campus. Patients were included in the study if they had been diagnosed with PD and were not experiencing severe cognitive decline. The Ethics Committees of the University of Haifa and the Rambam Health Care Campus approved the study. All participants provided written informed consent.

### Procedure

Patients arriving for a routine visit at the Movement Disorders Institute were screened for eligibility by the treating neurologist. Eligible patients were given a short explanation of the study procedures and invited to participate in a one-hour session consisting of answering questionnaires and assessments, described below, conducted by a trained research assistant. Data about the disease and pharmacological treatment were retrieved from electronic medical records.

### Study variables

Information on participants’ age, gender, years of education, and disease duration and disease severity was collected. To assess disease severity, we used the following variables, which are disease-specific indicators of the severity of PD, but each reflects a different aspect of the disease (motor symptoms, non-motor symptoms, and dopamine consumption): (1) the Unified Parkinson’s disease rating scale revised by the Movement disorders society (MDS-UPDRS), motor part score, to assess severity of motor signs of PD [34]; (2) the Non-Motor Symptoms Questionnaire score (NMSQ) [35], to assess severity of non-motor signs of PD; and (3) the Levodopa Equivalent Daily Dose (LEDD) [36], which provides an artificial summary of the total daily anti-Parkinsonian medications a patient is receiving [36, 37].

Health behaviors included utilization of rehabilitative treatments and physical activity level. Participants were asked to report whether and how frequently they utilize physical therapy, occupational therapy, and speech and language pathology treatments. The International Physical Activity Questionnaire–Short version (IPAQ-Short) [31] was used to assess physical activity. The IPAQ is a commonly used, self-report questionnaire of time spent in four categories of physical activity (i.e., vigorous, moderate, walking, and sitting) during the last seven days. Scores are interpreted as total physical activity in metabolic equivalent units (METS) in the last 7 days [31].

Patients’ activation was assessed with the Patient’s Activation Measure (PAM-13®, Insignia Health) [32]. The PAM-13 is a self-report, validated, licensed tool to measure a patient’s knowledge, skills, and confidence for self-management [32]. We used a validated, licensed Hebrew version of the PAM-13 supplied by Insignia Health (https://www.insigniahealth.com/products/pam-survey), which holds the copyrights to the questionnaire. The PAM-13 consists of 13 items, to which participants rate their agreement on a 4-point Likert scale. The score is transformed into a continuous 0–100 scale according to a licensed conversion table (Insignia Health) [33]. A patient’s overall score captures the extent to which they feel engaged and confident in managing their health conditions, with higher scores indicating stronger activation [32]. Based on their PAM-13 score, patients are divided into four ordinal levels of activation. Level 1 represents patients who tend to be passive and feel overwhelmed managing their own health, while level 4 represents patients who have the attitude, knowledge and skills that are important for engagement in health behaviors.

Health outcomes were evaluated across two domains: function, and HRQoL. Function was assessed with (1) the Frenchay Activities Index (FAI) [38], and (2) the 10 Meter Walk Test (10-MWT) for comfortable walking speed. Walking speed correlates with functional mobility and physical function in PwP and other populations [39–41]. Frenchay index and gait speed were standardized to mean 0 and SD 1 and then averaged to produce the dependent variable of functioning.

HRQoL was assessed using the PD Questionnaire–39 (PDQ-39) [42]. The questionnaire items measure frequency of experiencing difficulties across eight dimensions of daily living. The HRQoL construct was developed using two indicator variables: the emotions and cognition subscales of the PDQ-39. The theory-based selection of these specific subscales was further strengthened by factor analysis. We conducted a factor analysis using a Varimax rotation method that confirmed that all PDQ subscales constitute a single construct; Therefore, only the two subscales with the highest loadings were used: the PDQ Emotions and Cognition scales (0.82 and 0.80 respectively).

### Statistical analysis

Descriptive statistics were calculated to describe the study sample and to characterize utilization of rehabilitative treatments, physical activity, and patient activation levels. Nominal and ordinal variables are presented using frequency indices. Continuous variables are presented as mean ± standard deviation (SD) or as median and a range between quartiles 1 and 3, depending on the distribution of the variable.

For the walking-speed variable, a missing value for one participant was corrected using the mean of all participants.

Linear regression models were used to evaluate if health behaviors (physical activity and patient activation) were associated with functioning and HRQoL, controlling for demographics and disease severity. We used an iterative approach to developed our final model. First, we tested a model that included only demographic variables (sex, age, disease duration, years of education, social support (MSPSS score), co-morbidities (Charlson Index) and living with another person (indicating support at home); Second, we entered variables related to disease severity (NMSQ, MDS-UPDRS and LEDD); Third, we entered health behaviors (IPAQ and PAM), then we entered interactions between health behaviors and disease severity variables. Finally, a final reduced model was tested omitting non-significant variables. Variables included in the final model were selected using the backward method.

The level of statistical significance was determined to be *p* < 0.05 for all analyses. Analyses were performed using SAS version 9.4.

## Results

A total of 90 PwP were recruited, of whom 88 (58 men, 30 women, mean age 66.84 ± 8.8, mean Hoehn & Yahr stage 2.5 ± 0.8) had complete data and were included in the analyses. The majority of participants (84.1%) resided with someone else at home, suggesting they had family or caregiver support at home. Additionally, approximately one-third (33.3%) received assistance from social security, signifying a certain degree of limitation in their Activities of Daily Living (ADLs).

Table 1 presents sample demographics and disease-related characteristics.

**Table 1:** The sample demographics and disease-related characteristics (N = 88)

Characteristic	Mean ± SD or frequencies in %
Age (years)	66.84 ± 8.8
Male	65.9% (58)
Female	34.1% (30)
Education (years)	14.14 ± 3.25
Living with another person at home	84.1% (74)
Help from Social Security	33.3% (29)
Hoehn & Yahr stage	–
Stage 1	4.6% (4)
Stage 2	59.8% (52)
Stage 3	20.7% (18)
Stage 4	14.9% (13)
Stage 5	0

### Descriptive statistics of the measured variables

Table 2 presents descriptive statistics of disease severity, health behaviors, function, and HRQoL.

**Table 2:** Descriptive statistics of the study variables

Measured variables	Mean score ± SD/Median (min–max)
PAM (0–100)*	67.29 ± 13.67
IPAQ (METS-min/week)	1876.74 ± 2127.04
NMSQ (0–30)	10.66 ± 5.45
LEDD (mg/day)	763.86 ± 573.01
MDS-UPDRS motor part (0–132)	31 (1–94)
FAI (0–45)	25.61 ± 10.36
10-MWT (m/sec)	0.99 ± 0.26
PDQ cognitions (0–100)	26.89 ± 21.23
PDQ emotions (0–100)	25.98 ± 24.84

PAM, Patient Activation Measure; IPAQ, International Physical Activity Questionnaire; NMSQ, Non-Motor Symptoms Questionnaire; LEDD, Levodopa Equivalent Dose; MDS-UPDRS, Movement Disorders Society-Unified Parkinson’s disease rating scale; FAI, Frenchay Activities Index; 10-MWT, 10 Meter Walk Test; PDQ cognitions, Parkinson Disease Questionnaire, cognitions; PDQ emotions, Parkinson Disease Questionnaire, emotional well-being

*Numbers in parentheses for the PAM, NMSQ, MDS-UPDRS motor part, FAI, and PDQ indicate the range of scores for each of these assessments

Utilization of rehabilitative treatment was as follows: 14.8% (n = 13) attended physical therapy; 2.3% (n = 2), occupational therapy; 5.7% (n = 5), hydrotherapy; 2.3% (n = 2), speech and language pathology; and 2.3% (n = 2), psychology. Since utilization of healthcare services was almost negligible, this variable was not included in the regression analysis.

Overall, 8% of the participants were assigned PAM Level 1 (PAM ≤ 47), 10.2% were assigned PAM Level 2 (47 < PAM ≤ 55), 43.2% were assigned PAM Level 3 (55 < PAM ≤ 67), and 38.6% were assigned PAM Level 4 (PAM > 67).

### Correlations between study variables

A preliminary Pearson correlation analysis of the research variables (see Additional file 1) revealed significant correlations between the variables, ranging from r = 0.22 to r = 0.70. These results confirmed the relevance of the selected variables for the model.

### Multiple regression analyses for function

In the final iteration, multiple linear regression was used to test if IPAQ, PAM and MDS-UPDRS were significantly associated with function. A summary of each iteration model is presented in Table 3 and the complete results of the final model are presented in Table 4.

**Table 3:** Summary of linear regression models for associations with function

	N	R^2^adj	Model significance	IPAQ sig	PAM sig
Demographics (reduced model including disease duration and years of education)	85	0.11	F_(2,82)_ = 6.09, *p* = 0.003	–	–
Demographics + Disease severity	85	0.37	F_(5,79)_ = 10.82, *p* < 0.001	–	–
Demographics + disease severity + health behaviors	85	0.51	F_(7,77)_ = 13.37, *p* < 0.001	*p* < 0.001	*P* = 0.04
Demographics + disease severity + health behaviors + interactions	85	0.5	F_(13,71)_ = 7.36, *p* < 0.001	*p* < 0.001	*p* = 0.03
Final model (including MDS-UPDRS, PAM and IPAQ)	87	0.51	F_(3,83)_ = 31.23, *p* < 0.001	*p* < 0.001	*P* = 0.02

PAM, Patient Activation Measure; IPAQ, International Physical Activity Questionnaire; MDS-UPDRS, Movement Disorders Society Unified Parkinson’s disease rating scale

Disease severity was assessed using NMSQ, LEDD, and MDS-UPDRS, and health behaviors were measured using IPAQ total score and PAM score

**Table 4:** Linear regression analysis for the final model for function

Variable	DF	Standardized coefficient	Standard Error	*t* Value	Significance (*p*)
Intercept	1	0	0.07	0.18	0.86
MDS-UPDRS	1	− 0.41	0.01	− 5.14	< 0.0001
PAM	1	0.2	0.01	2.43	0.02
IPAQ	1	0.4	0.003	5.08	< 0.0001

PAM, Patient Activation Measure; IPAQ, International Physical Activity Questionnaire; MDS-UPDRS, Movement Disorders Society Unified Parkinson’s disease rating scale

The overall regression was statistically significant (R^2^adj = 0.51, F(3, 83) = 31.23, *p* < 0.001). IPAQ and PAM were found to be significantly associated with function (b = 0.41, *p* < 0.001; b = 0.2, *p* = 0.02 respectively). Disease severity, indicated by MDS-UPDRS, was also found to be significantly associated with function (b = − 0.4, *p* < 0.0001).

### Multiple regression analyses for HRQoL

In the final iteration, multiple linear regression was used to test if age, NMSQ, MDS-UPDRS, IPAQ, NMSQ × IPAQ, and MDS-UPDRS × IPAQ were significantly associated with HRQoL. A summary of each iteration model is presented in Table 5 and the complete results of the final model are presented in Table 6.

**Table 5:** Summary of linear regression models for associations with HRQoL

	N	R^2^adj	Model significance	IPAQ significance	PAM significance
Demographics (reduced model including age, MSPSS)	85	0.11	F_(2,85)_ = 6.25, *p* = 0.003	–	–
Demographics + disease severity	88	0.49	F_(5,82)_ = 17.44, *p* < 0.001	–	–
Demographics + disease severity + health behaviors	88	0.49	F_(7,80)_ = 12.67, *p* < 0.001	NS	Ns
Demographics + health behaviors + disease severity + interactions	88	0.53	F_(13,74)_ = 8.52, *p* < 0.001	Ns NMSQ × IPAQ: *p* = 0.02 MDS-UPDRS × IPAQ: *p* = 0.01	Ns
Final model (including age, IPAQ, NMSQ, MDS-UPDRS, NMSQ × IPAQ, MDS-UPDRS × IPAQ)	88	0.52	F_(6,81)_ = 16.98, *p* < 0.001	*p* = 0.03 NMSQ × IPAQ: *p* = 0.02, MDS-UPDRS × IPAQ: *p* = 0.01	Ns

PAM, Patient Activation Measure; IPAQ, International Physical Activity Questionnaire; NMSQ, Non-Motor Symptoms Questionnaire; MDS-UPDRS, Unified Parkinson’s disease rating scale

Disease severity was assessed using NMSQ, LEDD, and MDS-UPDRS, and health behaviors were measured using IPAQ total score and PAM score

**Table 6:** Linear regression analysis for the reduced model for HRQoL

Variable	DF	Standardized Coefficient	Standard Error	*t* Value	Significance (*p*)
Intercept	1	0	11.82	3.79	< 0.001
Age	1	0.19	0.18	2.50	0.02
NMSQ	1	− 0.57	0.33	− 6.58	< 0.001
MDS-UPDRS	1	− 0.12	0.13	− 1.39	0.17
IPAQ	1	0.19	0.07	2.28	0.03
NMSQ x IPAQ	1	0.19	0.01	2.30	0.02
MDS-UPDRS x IPAQ	1	− 0.23	0.01	− 2.63	0.01

PAM, Patient Activation Measure; IPAQ, International Physical Activity Questionnaire; NMSQ, Non-Motor Symptoms Questionnaire; MDS-UPDRS, Movement Disorders Society Unified Parkinson’s disease rating scale

The overall regression was statistically significant (R^2^_ad_ = 0.52, F(6, 81) = 16.98, *p* < 0.001). IPAQ was found to be significantly associated with HRQoL (b = 0.19, *p* = 0.03). Disease severity as indicated by the NMSQ but not MDS-UPDRS was significantly associated with HRQoL (b = − 0.57, *p* < 0.001). Age was significantly associated with HRQoL (b = 0.19, *p* = 0.02). The interactions NMSQ × IPAQ and MDS-UPDRS × IPAQ were also significantly associated with HRQoL (b = 0.19, *p* = 0.02; b = − 0.23, *p* = 0.01, respectively).

**Fig. 1:**
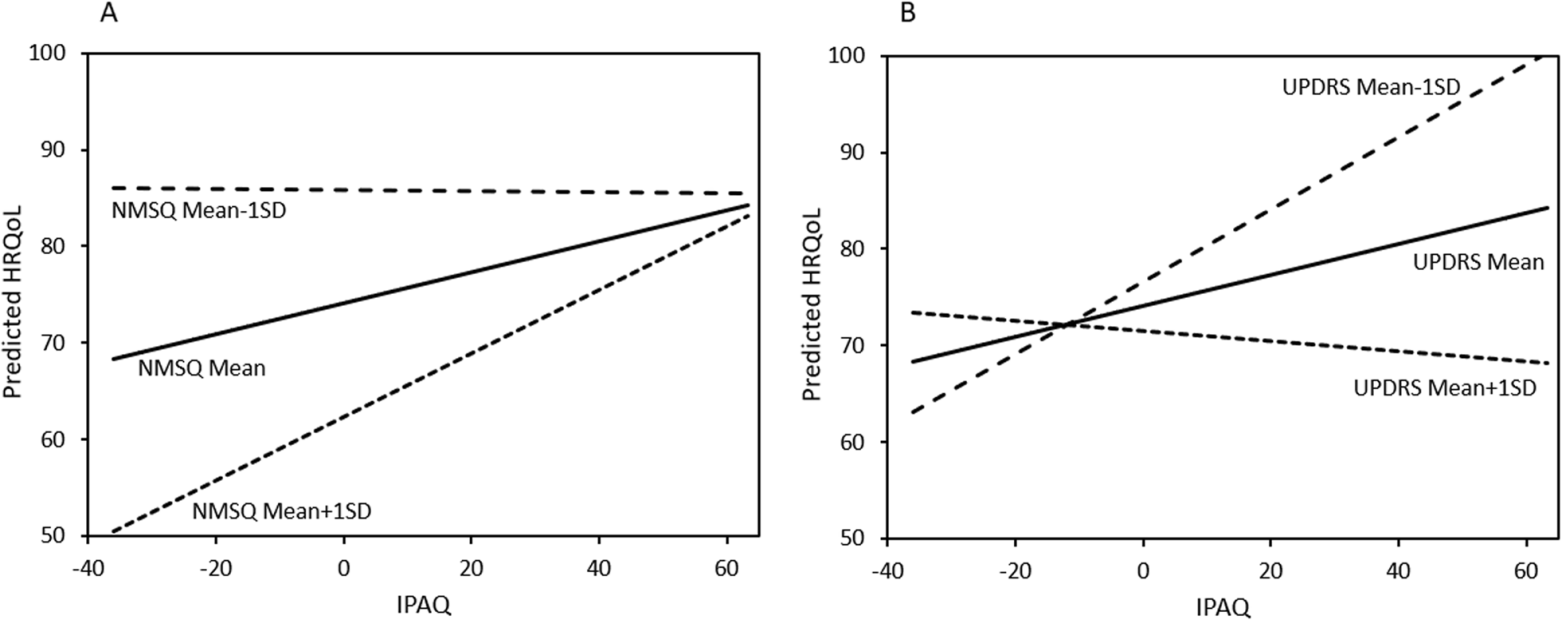
**A** Two-way interaction effect NMSQ*IPAQ on HRQoL (NMSQ levels are represented as mean (solid line), + 1SD (dashed line) and -1SD (spaced dashed line) slopes). IPAQ, International Physical Activity Questionnaire; NMSQ, Non-Motor Symptoms Questionnaire; HRQoL, Health-related quality of life; SD, standard deviation and **B** Two-way interaction effect MDS-UPDRS*IPAQ on HRQoL (MDS-UPDRS levels are represented as mean (solid line), + 1SD (dashed line) and -1SD (spaced dashed line) slopes). IPAQ, International Physical Activity Questionnaire; HRQoL, Health-related quality of life; MDS-UPDRS, Movement Disorders Society Unified Parkinson’s disease rating scale; SD, standard deviation

The interactions effects found showed that the strength of the association between IPAQ and HRQoL is a function of the NMSQ or MDS-UPDRS scores. It also showed that the direction of the interactions is opposite such that the association between IPAQ and HRQoL is stronger among people with higher NMSQ scores (i.e., more non-motor symptoms; Fig. 1A), while the association is stronger among people with lower MDS-UPDRS scores (i.e., fewer motor symptoms) (Fig. 1B).

## Discussion

This study provides information on PwPs’ self-reported engagement in PD-related health behaviors. We found that many patients do not utilize rehabilitative treatments. For example, only 14.8% of PwP in our study utilized physical therapy. This is in the lower range of rates previously reported in other countries, which ranged for physical therapy from 14% in the United States to 60% in the Netherlands [18, 43–45]. Among the services on which participants reported, physical therapy had the highest utilization rate, while hydrotherapy, occupational therapy, and speech and language pathology had substantially lower rates. Our findings may be explained by barriers at the individuals and healthcare-system levels that impede PwPs’ utilization of these services, such as patients’ or physicians’ low awareness of the benefits of rehabilitation treatments, low availability and accessibility of rehabilitation services [46], and possibly low health insurance literacy [47]. Furthermore, at the healthcare-system level, there is a notable absence of practical framework for the delivery of rehabilitative treatments in progressive chronic disease such as PD.

The IPAQ Research Committee [48] suggests a cutoff value of 600 MET-min/week to distinguish between people engaged in light and moderate levels of physical activity and 3000 MET-min/week to distinguish between people engaged in moderate and vigorous levels of activity. Accordingly, our sample was engaged in physical activity at a moderate level (mean ± SD score = 1876.74 ± 2127.04 MET-min/week). This is similar to the level of physical activity previously reported in PwP in an Australian cohort (1823.6 ± 1693.6 MET-min/week) [49].

Average PAM scores and distributions across PAM levels were similar to those reported for other chronic diseases, such as cancer, diabetes mellitus, and asthma [49]. For example, Hibbard et al. [50] reported a mean PAM score of 64.2 in these populations, in comparison to 67.3 in our study. Most of our participants were at level 3 or 4, indicating high levels of patient activation. It is possible that this finding is biased by the fact that our participants were recruited from a Movement Disorder Institute and thus were already relatively active in managing their care and might have knowledge about their disease. The discrepancy between the moderate-high level of activation and the very low engagement in rehabilitative treatments reveals heterogeneity across the dimensions of health behaviors. Specifically, it shows that a person may have the knowledge, skills, and confident to self-manage their disease, yet fail to take actual steps to engage in health behavior actions. Thus, these findings underscore that the construct of health behaviors in PD is complex and requires multidimensional assessment.

The study results show associations between health behaviors (including physical activity and patient activation) and function. These behaviors, in combination with motor symptom severity, explain approximately 50% of the variance in function. Findings also show that physical activity (but not patient activation) and its interactions with motor and the non-motor symptoms of PD are associated with HRQoL. In combination with age and non-motor symptoms, physical activity explains approximately 50% of the variance in HRQoL. This structure of relationships was not previously demonstrated.

Higher levels of physical activity have been associated with higher function and quality of life in other chronic diseases [51–53], and specifically in PD [30, 54]. Our results highlight the role of disease severity in the association between physical activity and HRQoL. Physical activity has a greater contribution to HRQoL in patients with more non-motor symptoms relative to those with fewer non-motor symptoms. In contrast to the effects of motor symptoms, the presence of non-motor symptoms does not seem to limit the potential of physical activity to improve HRQoL. The clinical implications of this finding supports the importance of engaging in physical activity even for patients with advanced non-motor symptoms. However, greater severity of motor symptoms may diminish the positive effect of physical activity on HRQoL. The interaction effect of severity may be unique to PD, as the disease is characterized by a very wide spectrum of debilitating symptoms across many body systems.

Specific novel insights gained in our study refer to the role of patient activation. Prior literature on chronic diseases associated higher patient activation with reduced disease-related symptoms, and with higher HRQoL and function [23, 55, 56]. To the best of our knowledge, this is the first study to report an association between patient activation and function in PwP. In a recent study [57], PwP with higher levels of patient activation were less susceptible to the negative impact of COVID-19-imposed social distancing [57]. The finding of the current study joins these findings and suggests that patient activation has a positive impact on function in PwP. Patient activation was not associated with HRQoL. The measurement of patient activation mainly relates to patients’ readiness to take action and, to a lesser degree, to aspects of patients’ emotional coping. This may explain why PAM was associated with function but not with HRQoL.

Policy implications: this study draws the attention to health behaviors of PwP. Our findings emphasize the connection between physical activity and patient engagement and function. This highlights the need for policymakers to make physical activity opportunities more available and accessible for PwP within the healthcare system. This trend aligns with the growing recognition of physical activity as a key aspect of managing various chronic conditions. Furthermore, our study reveals that rehabilitative healthcare services are being underutilized by PwP, despite the known benefits these services offer for health and quality of life. To increase the utilization of rehabilitative treatments, there is a need to address not only aspects related to patients but also healthcare policy. Healthcare organizations could adopt a proactive approach by providing educational materials on the benefits of rehabilitative care, encouraging general practitioners to refer PwP to such services, and actively engaging PwP in specialized programs like exercise groups within community clinics. Informative brochures about the benefits of rehabilitative care and information about what the patient needs to do in bureaucratic matters to receive treatment could be distributed to patients by general practitioners, neurologists, allied health professionals, or through patient organizations (such as the Israel Parkinson Association). The value of knowledge and information for treatment utilization and adherence was supported in research among PwP [58, 59]. As to encouraging general practitioners to refer PwP to rehabilitative treatments, we suggest that the utilization of these services may be considered a quality indicator of the treatment of PwP. This is aligned with current guidelines and improve utilization rates. Health maintenance organizations (HMOs) should enhance opportunities for engaging in rehabilitative activities. This necessitates establishing specific services like group training and multidisciplinary rehabilitation within the community.

Our study has several limitations. Participants’ cognitive status was informally evaluated by the treating neurologist. Utilization of rehabilitative treatments was not included in the analysis due to very low rates of utilization. Level of physical activity relied on responses to a self-report questionnaire (IPAQ). Future research may use objective measures such as activity monitors. In addition, previous physical activity was not documented. Future research could encompass long-term physical activity to gain a more thorough understanding of how it relates to function and HRQoL.

## Conclusions

The results of this study showed underutilization of an important domain of health behaviors that have potential benefits for PwP, i.e., treatments by health professionals. The associations found support the hypothesis that health behaviors are important for maintaining function and HRQoL in PwP. They also reveal that motor and non-motor symptoms have a differential contribution to the association between physical activity and quality of life.

### Practice implications

Our study has clinical implications. Patients and physicians should be made aware of the scientific evidence supporting the positive impacts of rehabilitative treatments on disease severity, function, and HRQoL in PwP, in order utilization. In addition, our findings support facilitation of patient activation as a tool to promote HRQoL and maintain function, through the development and implementation of support programs that provide access to knowledge and training in self-management skills.

### Supplementary Information

**Additional file 1**. Correlations between study variables.

## Data Availability

The data analyzed during the current study are available at: https://zenodo.org/deposit/5931646

## Abbreviations

PD: Parkinson’s disease
PwP: People living with PD
HRQoL: Health-related quality of life
MDS-UPDRS: Unified Parkinson’s disease rating scale
NMSQ: Non-motor symptoms questionnaire score
LEDD: Levodopa equivalent daily dose
IPAQ-Short: International physical activity questionnaire–short version
PAM-13: Patient’s activation measure
FAI: Frenchay activities index
10-MWT: 10 meter walk test
PDQ-39: PD questionnaire–39
SD: Standard deviation
MSPSS score: Multidimensional scale of perceived social support
