# Supplementary material for: Association of health behaviors with function and health-related quality of life among patients with Parkinson’s disease
Source: Isr J Health Policy Res. 2024 Jan 3;13:2. doi: 10.1186/s13584-023-00588-3 (PMC10763356; doi:10.1186/s13584-023-00588-3)
Supplement: Supplementary file 1 — Additional file 1. Correlations between study variables. [file 13584_2023_588_MOESM1_ESM.docx]

| **Observed measures** | | IPAQ | PAM-13 | NMSQ | FAI | 10-MWT | LEDD | MDS-UPDRS | PDQ emotions | PDQ cognitions |
| --- | --- | --- | --- | --- | --- | --- | --- | --- | --- | --- |
|  | IPAQ | — |  |  |  |  |  |  |  |  |
|  | PAM-13 | **0.327^**^** | — |  |  |  |  |  |  |  |
|  | NMSQ | **−0.332^**^** | **−0.481^***^** | — |  |  |  |  |  |  |
|  | FAI | **0.529^***^** | **0.413^***^** | **−0.537^***^** | — |  |  |  |  |  |
|  | 10-MWT | **0.447^***^** | **0.415^***^** | **−0.399^***^** | **0.701^***^** | — |  |  |  |  |
|  | LEDD | −0.099 | **−0.229^*^** | **0.337^**^** | **−0.284^**^** | **−0.293^**^** | — |  |  |  |
|  | MDS-UPDRS | −0.163 | **−0.327^**^** | **0.418^***^** | **−0.560^***^** | **−0.432^***^** | **0.225^*^** | — |  |  |
|  | PDQ emotions | **−0.364^***^** | **−0.328^**^** | **0.547^***^** | **−0.368^***^** | **−0.243^*^** | 0.167 | **0.218^*^** | — |  |
|  | PDQ cognitions | **−0.255^*^** | **−0.428^***^** | **0.667^***^** | **−0.507^***^** | **−0.272^*^** | 0.205 | **0.375^**^** | **0.588**** | — |

* p < 0.05, ** p < 0.01, *** p < 0.001.
IPAQ, International Physical Activity Questionnaire; PAM, Patient Activation Measure; NMSQ, Non-Motor Symptoms Questionnaire; FAI, Frenchay Activities Index; 10-MWT, 10 Meter Walk Test; LED, Levodopa Equivalent Dose; UPDRS, Unified Parkinson’s disease rating scale; PDQ cognitions, Parkinson Disease Questionnaire, cognitions; PDQ emotions, Parkinson Disease Questionnaire, emotional well-being.
